# Refining the pathway of carbide insertion into the nitrogenase M-cluster

**DOI:** 10.1038/ncomms9034

**Published:** 2015-08-11

**Authors:** Jared A. Wiig, Yilin Hu, Markus W. Ribbe

**Affiliations:** 1 Department of Molecular Biology and Biochemistry, University of California, Irvine, California 92697-3900, USA; 2 Department of Chemistry, University of California, Irvine, California 92697-2025, USA

## Abstract

Carbide insertion plays a pivotal role in the biosynthesis of M-cluster, the cofactor of nitrogenase. Previously, we proposed a carbide insertion pathway involving methyltransfer from SAM to a FeS precursor and hydrogen abstraction from this methyl group that initiates the radical-based precursor maturation. Here we demonstrate that the methyl group is transferred to a precursor-associated sulfur before hydrogen abstraction, thereby refining the initial steps of the carbide insertion pathway.

## Abstract (web summary)

Carbide insertion plays a pivotal role in the biosynthesis of M-cluster, the cofactor of nitrogenase. Here the authors further define the pathway for interstitial carbide atom insertion, showing that the SAM-derived methyl group is transferred to a FeS precursor sulfur before hydrogen abstraction via an SN2-type reaction.

## Graphical abstract

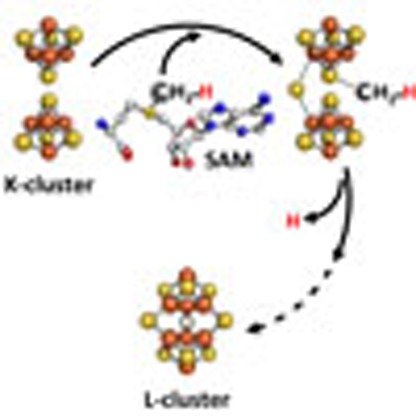

Molybdenum (Mo) nitrogenase catalyses the reduction of nitrogen (N_2_) to ammonia (NH_3_) under ambient conditions at its active cofactor center^1^,^2^. Designated the M-cluster (Fig. 1), this complex metallocofactor has a [MoFe_7_S_9_C] core that can be viewed as [MoFe_3_S_3_] and [Fe_4_S_3_] subclusters bridged by three μ_2_-sulfide atoms and one μ_6_-carbide ion (C^4−^) in between, and it is further coordinated by an organic homocitrate moiety at its Mo end^3^,^4^,^5^,^6^. Since its discovery, the μ_6_-carbide at the centre of the M-cluster has attracted considerable attention because of its potential relevance to the structure and function of nitrogenase. More recently, a radical SAM-dependent pathway was identified for the incorporation of carbide into the M-cluster, generating new interest in elucidating the mechanistic details of this key event in the biosynthesis of M-cluster^7^,^8^.

Biosynthesis of the M-cluster involves the formation of a FeS core on a radical *S*-adenosyl-L-methionine (SAM) enzyme, NifB; and the subsequent maturation of this core into a mature M-cluster on a scaffold protein, NifEN^7^,^8^,^9^,^10^,^11^. Previously, we demonstrated through studies of a NifEN-B fusion protein that, in the presence of SAM, NifB was capable of coupling a [Fe_4_S_4_]-like cluster pair (designated the K-cluster, or the FeS precursor) into an [Fe_8_S_9_C] cluster (designated the L-cluster, or the FeS core) concomitant with the incorporation of a carbide and a ‘9^th^’ sulfur^7^. Further, we identified the methyl group of SAM as the source of carbide and established hydrogen abstraction as the initial step to process this methyl group into a carbide ion^8^. Together, these observations have led to the proposal of a carbide insertion pathway (Fig. 1), which involves: (i) the transfer of a methyl group from SAM to the K-cluster on NifB; (ii) the abstraction of a hydrogen atom from this methyl group by a 5′-deoxyadenosyl radical (5′-dA·); and (iii) further deprotonation/dehydrogenation of the resultant methylene radical into a carbide ion concomitant with the insertion of the ‘9^th^’ sulfur and the radical-based rearrangement and coupling of the two [Fe_4_S_4_]-like modules of the K-cluster into an [Fe_8_S_9_C] L-cluster^7^,^8^. The L-cluster is then matured into an M-cluster on NifEN following NifH-mediated insertion of Mo and homocitrate and transferred to its target binding site in NifDK, completing the assembly of M-cluster^9^,^10^. While attractive, certain details of this model, such as where the methyl group is initially attached on transfer and whether methyltransfer occurs before hydrogen abstraction, have remained unclear.

Here we show that the methyl group of SAM is transferred to a K-cluster-associated sulfur before hydrogen abstraction from this group takes place. This observation establishes the sequence of events during the initial phase of carbide insertion and reveals an interesting SN2-type reaction that involves direct methyltransfer to a metallosulfur cluster.

## Results

### Defining the initial point of methyl attachment

To pinpoint the entry point of the methyl group into the pathway of carbide insertion, NifEN-B was first incubated with SAM to allow methyltransfer to occur and subsequently treated by acid and analysed for methyl-bound species. Consistent with mobilization of the methyl group of SAM and abstraction of hydrogen from this methyl group by 5′-dA·, high-performance liquid chromatography (HPLC) analysis revealed that SAM was cleaved into *S*-adenosyl-L-homocysteine (SAH) and 5′-deoxyadenosine (5′-dAH) on incubation with NifEN-B in the presence of a reductant, dithionite (Fig. 2a, trace 3). Substitution of [methyl-*d*_*3*_] SAM for SAM in this incubation mixture resulted in the same HPLC profile as that of the cleavage of SAM, although 5′-dAD was generated in place of 5′-dAH as a result of hydrogen abstraction from the deuterium-labelled methyl group (Fig. 2a, trace 4). Interestingly, when the incubation mixture comprising SAM, NifEN-B and dithionite were quenched by acid, a new, volatile product could be detected by gas chromatograph (GC) (Fig. 2b, black) and identified further by gas chromatography–mass spectrometry (GC–MS) analysis as methanethiol (CH_3_SH) based on a mass-to-charge (*m*/*z*) ratio of 47 (Fig. 2b, inset). Likewise, when the incubation mixture comprising [methyl-*d*_*3*_] SAM, NifEN-B and reductant was quenched by acid, a volatile product with identical retention time to that of methanethiol could be detected on the same GC column (Fig. 2c, black). GC–MS analysis identified this product as methane-*d*_*3*_-thiol (CD_3_SH) based on an *m*/*z* ratio of 50 (Fig. 2c, inset), confirming that the methyl group in methanethiol originated from the methyl group of SAM.

**Figure 1:**
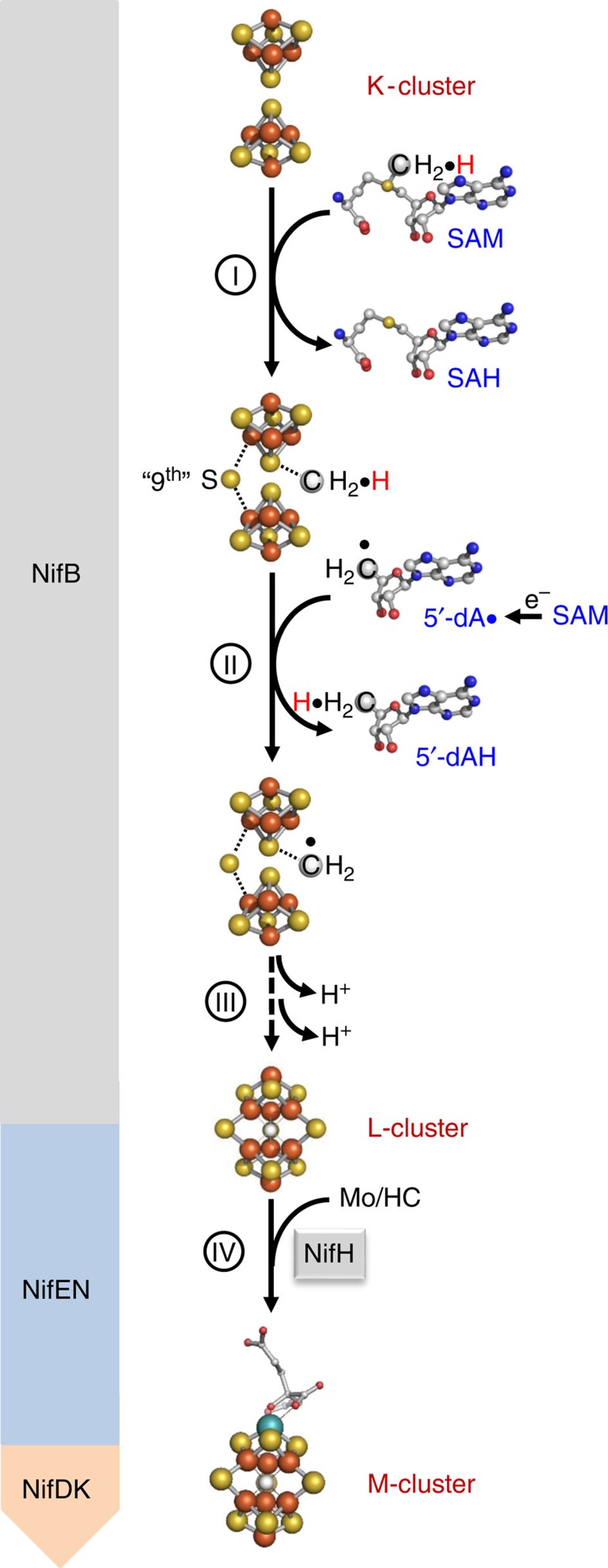
Proposed carbide insertion pathway. It begins with the transfer of a methyl group from one equivalent of SAM to the K-cluster (I) and abstraction of a hydrogen atom from the methyl group by a 5-dA· radical that is generated upon homolytic cleavage of a second equivalent of SAM (II). The process continues with further dehydrogenation/deprotonation of the methylene radical concomitant with its incorporation into the L-cluster as an interstitial carbide atom (III). The L-cluster is further matured into an M-cluster on insertion of Mo and homocitrate (IV). The Nif proteins hosting events I–IV during the assembly of M-cluster are indicated in the figure. Atoms of the clusters are coloured as follows: Fe, orange; S, yellow; C, gray; Mo, cyan. HC, homocitrate.

**Figure 2:**
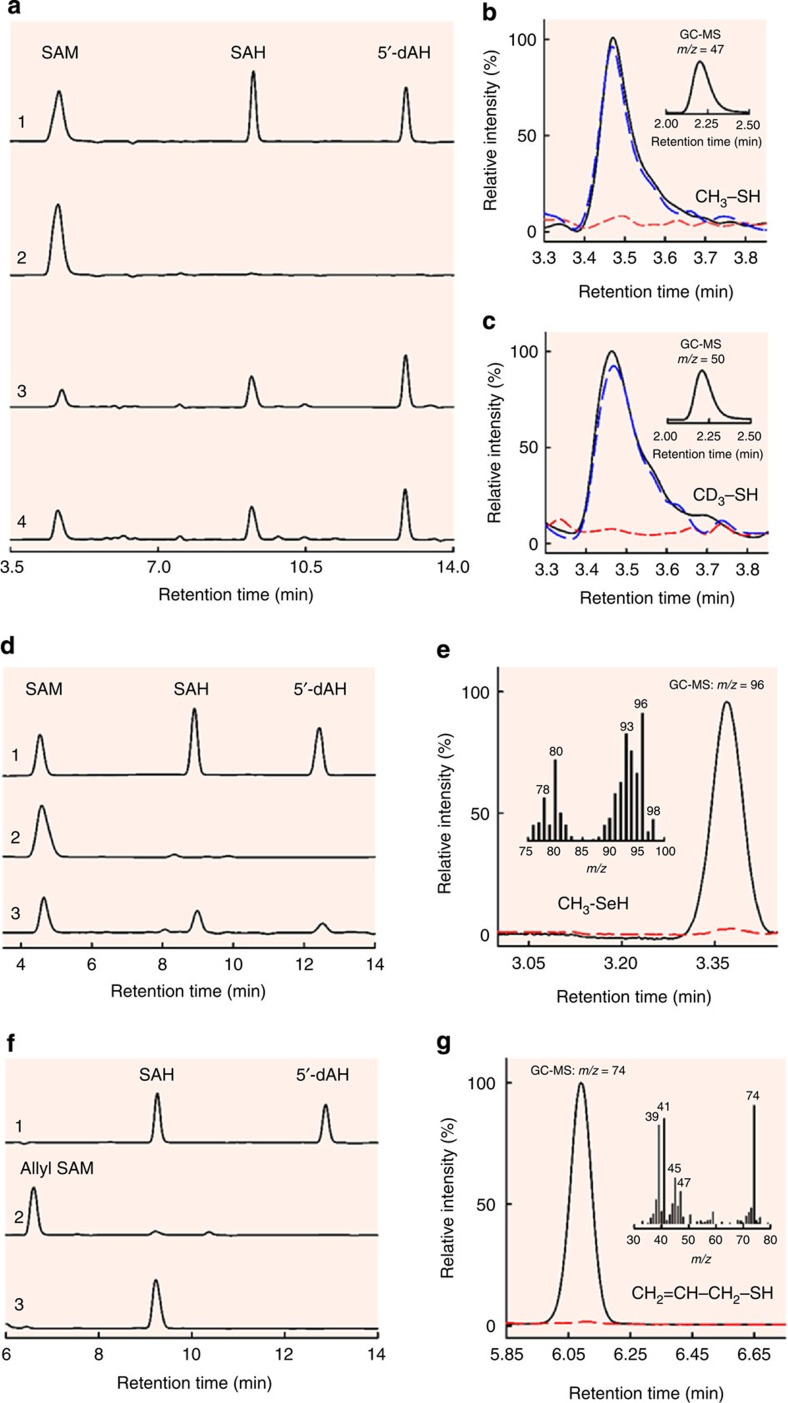
HPLC analyses of cleavage products of SAM/analogs and GC/GC–MS analyses of acid-quenched products upon incubation of NifEN-B/NifEN-B^FeSe^ with SAM/analogues. (**a**) HPLC elution profiles of (1) SAM, SAH and 5′-dAH standards; (2) SAM alone; (3) SAM and (4) [methyl-*d*_*3*_] SAM in the presence of reduced NifEN-B. (**b**,**c**) GC–MS (inset) and GC analyses of acid-quenched incubation mixtures containing SAM (**b**) or [methyl-*d*_*3*_] SAM (**c**), and NifEN-B in the reduced (black), oxidized (red) or re-reduced (blue) state. The acid-quenched products were identified as methanethiol (**b**, inset) and methane-*d*_*3*_-thiol (**c**, inset) based on their GC retention times and respective *m*/*z* ratios of 47 and 50. (**d**) HPLC elution profiles of (1) SAM, SAH and 5′-dAH standards; (2) SAM alone; and (3) SAM in the presence of reduced NifEN-B^FeSe^. (**e**) GC–MS full scan (inset) and SIM (*m*/*z*=96) analyses of acid-quenched incubation mixtures containing reduced NifEN-B^FeSe^ in the presence (black) and absence (red) of SAM. The acid-quenched product was identified as methylselenol based on its GC–MS retention time and fragmentation pattern. (**f**) HPLC elution profiles of (1) SAH and 5′-dAH standards; (2) allyl SAM alone; and (3) allyl SAM in the presence of reduced NifEN-B. (**g**) GC–MS full scan (inset) and SIM (*m*/*z*=74) analyses of acid-quenched incubation mixtures containing reduced NifEN-B in the presence (black) and absence (red) of allyl SAM. The acid-quenched product was identified as allylthiol based on its GC–MS retention time and fragmentation pattern.

The observation of attachment of this SAM-derived methyl group to an acid-labile sulfur atom is exciting, as it provides the first indication that the methyl group of SAM is transferred to a sulfur atom of a FeS cluster, such as one of the [Fe_4_S_4_] modules of the K-cluster in our proposed model (see Fig. 1). To further investigate the source of sulfur in the methanethiol product, NifEN-B was first treated with an iron chelator to remove its endogenous FeS clusters and then reconstituted with FeCl_3_/Na_2_Se or FeCl_3_/Na_2_S. When SAM was incubated with the Fe/Se-reconstituted NifEN-B protein (designated NifEN-B^FeSe^) in the presence of dithionite, it was cleaved into SAH and 5′-dAH (Fig. 2d, trace 3)—the same products generated from SAM cleavage by the as-isolated NifEN-B or the Fe/S-reconstituted NifEN-B protein (designated NifEN-B^FeS^)—although the efficiency of SAM cleavage by NifEN-B^FeSe^ was lower than that by NifEN-B or NifEN-B^FeS^. When this incubation mixture was quenched by acid, a volatile product was detected by GC analysis and identified as methylselenol (CH_3_SeH) by GC–MS analysis based on a *m*/*z* ratio of 96, as well as a characteristic fragmentation pattern of this compound (Fig. 2e, inset). This result firmly establishes the S atom of a FeS cluster as the point of methyl group attachment and points to the possibility that this methyltransfer step occurs via an SN2-type reaction before the abstraction of a hydrogen atom from the methyl group.

### ‘Uncoupling’ methyltransfer and hydrogen abstraction

To investigate the sequence of events between methyltransfer and hydrogen abstraction, the two events were ‘uncoupled’ through substitution of allyl SAM, an analogue containing an allyl group (–CH_2_–CH=CH_2_) in place of a methyl group (–CH_3_), for SAM in the incubation mixture containing NifEN-B and reductant. HPLC analysis demonstrated that allyl SAM was cleaved into SAH on incubation with NifEN-B and reductant, indicating that allyl transfer occurred during this process; however, no 5′-dAH could be detected in this case, suggesting that allyl SAM was incapable of undergoing homolytic cleavage to give rise to a 5′-dA· radical for hydrogen abstraction from the allyl group (Fig. 2f, trace 3). When the incubation mixture comprising allyl SAM, NifEN-B and dithionite were quenched by acid and extracted into dichloromethane (DCM), a volatile product was detected by GC analysis (Fig. 2g, black) and identified as allylthiol (CH_2_=CH–CH_2_–SH) based on an *m*/*z* ratio of 74 and a characteristic fragmentation pattern of this compound (Fig. 2g, inset). This observation provides compelling evidence that the transfer of methyl group occurs before the abstraction of hydrogen by a 5′-dA· radical while further implying that the initial methyltransfer from SAM to the S atom of Fe–S cluster follows an SN2-type mechanism.

**Figure 3:**
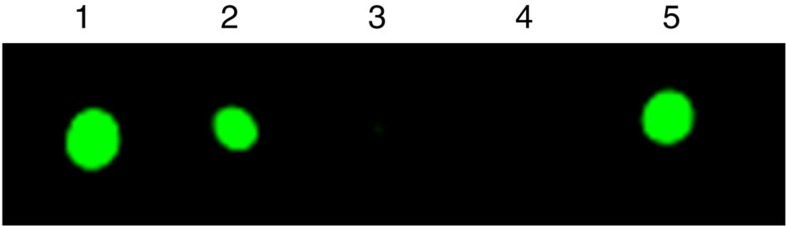
Capture of NifEN-B proteins in different redox states on affinity (IMAC) resin after incubation with [methyl-^14^C] SAM. NifEN-B samples were as following: (1) reduced by excess dithionite; (2) reduced by dithionite, followed by removal of excess dithionite; (3) oxidized by IDS; (4) oxidized by IDS and then re-reduced by dithiothreitol; and (5) oxidized by IDS and then re-reduced by dithionite.

Interestingly, while an SN2-type methyltransfer does not require the SAM-binding FeS cluster on NifEN-B to be present in a reduced state, the formation of methanethiol (Fig. 2b, red) or methane-*d*_*3*_-thiol (Fig. 2c, red) was completely abolished when NifEN-B was oxidized by indigo disulfonate (IDS), but could be restored on re-reduction of the NifEN-B protein by dithionite (Fig. 2b,c, blue). Consistent with this observation, radiolabelling experiments demonstrated the transfer of methyl when NifEN-B was reduced by dithionite (Fig. 3, lanes 1, 2 and 5) and the absence of this event when this protein was oxidized by IDS (Fig. 3, lane 3) or reduced by a weak reductant, dithiothreitol (DTT) (Fig. 3, lane 4). Together, these observations suggest that the K-cluster needs to be poised at a certain redox potential to render its associated sulfur atom more nucleophilic for the transfer of methyl group from SAM via an SN2-type nucleophilic substitution.

## Discussion

While the post-hydrogen-abstraction events along the carbide insertion pathway are yet to be explored, the current work has established the first two steps in this process as an SN2-type methyltransfer from SAM to the sulfur atom of the K-cluster, followed by hydrogen abstraction of this SAM-derived methyl group by a 5′-dA· radical. Given that the endogenous FeS clusters could be removed from NifEN-B and readily reconstituted by Fe/S or Fe/Se (see Fig. 2d,f) through a method specific for FeS cluster formation, the ‘9^th^’ sulfur that is required along with the carbide for the coupling and rearrangement of the two [Fe_4_S_4_]-like cluster modules of K-cluster into an [Fe_8_S_9_] L-cluster seems to be associated with the FeS cluster instead of the protein environment. One appealing scenario is that the ‘9^th^’ sulfur is a ‘dangling’ sulfur attached externally to one Fe atom of the K-cluster, which serves as the initial point of methyl attachment. Such ‘dangling’ sulfurs have been observed in the cases of other radical SAM-dependent enzymes such as RimO, MiaB and HydG^12^,^13^, as well as the radical enzyme (*R*)-2-hydroxyisocaproyl-CoA dehydratase^14^, which enable a variety of important FeS chemistry in biological systems. Strategic labelling of this sulfur by Se may shed light on the insertion of this atom that is intertwined with the insertion of carbide, which could facilitate further refinement of the assembly pathway of M-cluster.

## Methods

Unless noted otherwise, all chemicals and reagents were obtained from Fisher Scientific or Sigma-Aldrich. All proteins were handled under argon in an anaerobic chamber containing <4 p.p.m. O_2_.

### Cell growth and protein purification

*Azotobacter vinelandii* was grown at 30 °C in 180-l batches in a 200-l fermenter (New Brunswick Scientific) in Burke’s minimal medium supplemented with 2 mM ammonium acetate. Growth rates were monitored via cell density at 436 nm using a Spectronic 20 Genesys spectrometer (Spectronic Instruments). On ammonia depletion, cells were allowed to de-repress for 3 h before being harvested via a flow-through centrifugal harvester (Cepa). The cell paste was washed with 50 mM Tris–HCl (pH 8.0) buffer before it was frozen and stored at −80 °C. Published methods were adapted to the purification of His-tagged NifEN-B^7^.

### Radiolabel assays with [methyl-^14^C] *S*-adenosyl-L-methionine

Five different reactions were assembled, each containing, in a total volume of 32 μl, 100 mM Tris–HCl (pH 8.0), 250 μM [methyl-^14^C] SAM (American Radiolabeled Chemicals, Inc.) and NifEN-B: (i) in the as-isolated state, where an excess (10 mM) Na_2_S_2_O_4_ was supplied; (ii) in a reduced state, where excess Na_2_S_2_O_4_ was removed from the as-isolated NifEN-B (see i) via gel filtration with Sephadex G-25 fine resin (GE Healthcare); (iii) in an oxidized state, where Na_2_S_2_O_4_-free NifEN-B was oxidized with excess IDS, followed by the removal of excess IDS via AG1-X8 resin (Bio-Rad); (iv) in a weakly reduced state, in which oxidized NifEN-B was re-reduced by the addition of 5 mM DTT; and (v) in a re-reduced state, where the Na_2_S_2_O_4_-free NifEN-B (see ii) was first oxidized by IDS and then re-reduced by the addition of 10 mM Na_2_S_2_O_4_. All reactions were incubated for 30 min at 25 °C with intermittent mixing before being passed over Immobilized-metal affinity chromatography (IMAC) sepharose resin (10 μl packed volume; GE Healthcare) that was equilibrated with a buffer containing 25 mM Tris–HCl (pH 8.0), 10% glycerol and 500 mM NaCl. The IMAC sepharose resin was washed three times with 700 μl buffer containing 25 mM Tris–HCl (pH 8.0), 10% glycerol, 500 mM NaCl and 40 mM imidazole. The IMAC sepharose samples were then re-suspended in the wash buffer and applied directly onto a piece of Whatman 3-MM chromatography paper. The blots were dried and exposed to a GE Healthcare Storage Phosphor Screen GP (20 × 25 cm) for 48 h before imaging on a GE Healthcare Typhoon Trio^+^ variable mode imager.

### Selenium labelling experiment of NifEN-B

To generate selenium (Se)-labelled clusters on NifEN-B, the protein was first treated by an iron chelator that removed the endogenous iron–sulfur (FeS) cluster species from the protein, followed by re-isolation of the apo NifEN-B from the mixture. Specifically, 18 μM NifEN-B was incubated with 20 mM bathophenanthroline disulfonate in the presence of 2 mM Na_2_S_2_O_4_, 25 mM Tris–HCl (pH 8.0) and 10% glycerol for 30 min with gentle stirring. Subsequently, bathophenanthroline disulfonate was removed from the protein by passing the incubation mixture via a 10 ml packed Q-Sepharose Fast Flow column (GE Healthcare). The resultant apo NifEN-B was nearly colourless, exhibited no signal in electron paramagnetic resonance spectroscopy, and displayed no capability to serve as a precursor source in the L-cluster maturation assay^7^, suggesting that its endogenous FeS clusters have been removed.

The apo NifEN-B protein was chemically reconstituted with [Fe_4_S_4_] or [Fe_4_Se_4_] clusters following a procedure adapted from a previously published method^15^. First, Na_2_S_2_O_4_ was removed from NifEN-B via gel filtration with Sephadex G-25 fine resin. Then, 10 mM DTT was supplement to a solution containing 17 μM NifEN-B, 25 mM Tris–HCl (pH 8.0), 5% glycerol and 250 mM NaCl, and incubated on ice with gentle stirring for 10 min. Next, FeCl_3_ was slowly added to achieve a final concentration of 1 mM and the reaction was allowed to incubate for 5 min. Subsequently, Na_2_S or Na_2_Se was added to the reaction over a time period of 10 min to achieve a final concentration of 1 mM. The reaction was incubated in an ice water bath with gentle stirring for 1 h before being passed through the IMAC sepharose resin. The reconstituted NifEN-B protein that was bound to the IMAC sepharose resin was washed three times with four column volumes of a buffer containing 25 mM Tris–HCl (pH 8.0), 2 mM Na_2_S_2_O_4_, 10% glycerol, 500 mM NaCl and 40 mM imidazole and subsequently eluted in a buffer containing 25 mM Tris–HCl (pH 8.0), 2 mM Na_2_S_2_O_4_, 10% glycerol, 500 mM NaCl and 250 mM imidazole. The successful reconstitution of apo NifEN-B with [Fe_4_S_4_] clusters (designated NifEN-B^FeS^) or [Fe_4_Se_4_] clusters (designated NifEN-B^FeSe^) was confirmed by electron paramagnetic resonance analysis and cluster maturation assay^7^. NifEN-B^FeSe^ was 40% active relative to NifEN-B in the cluster maturation assays.

### Generation of allyl SAM

Allyl SAM, which contains an allyl group (CH_2_=CH–CH_2_–) in place of a methyl group (CH_3_–), was generated via an alkylation reaction following a procedure adapted from previously published methods^16^,^17^, in which 25 mg SAH was dissolved in 200 μl of a chilled acid solution consisting of formic acid and acetic acid at 1:1 ratio. Next, 80 μl of 3-bromo-1-propene was added and the reaction was incubated with stirring for 96 h before addition of 2 ml H_2_O to quench the reaction. The aqueous phase was extracted three times with 2 ml diethyl ether. Then, the lower, aqueous phase was lyophilized to dryness and dissolved in 300 μl H_2_O with 0.02% Trifluoroacetic acid (TFA) before allyl SAM was purified by HPLC using an Agilent 1200 Series HPLC system (Santa Clara, CA) equipped with a Supelcosil LC-18-T column (15 cm × 4.6 mm, 5-μM particle size). The column was equilibrated with 98% buffer A (50 mM KH_2_PO_4_, pH 6.6) and 2% buffer B (100% methanol). Following each 20-μl injection, the column was subjected to a linear gradient of 2–15% buffer B over 10 min, a linear gradient of 15–80% buffer B over 1 min, and an isocratic flow of 80% buffer B for 3 min. The flow rate of buffer was 1 ml min^−1^ throughout each run and the column was maintained at 25 °C. The column was prepared for subsequent sample injections by applying a gradient of 80–2% buffer B over 1 min, followed by an isocratic flow of 2% buffer B for 2 min. Under these conditions, allyl SAM was eluted between 3.4 and 4.2 min based on ultraviolet monitoring at 254 nm and confirmed to have a mass-to-charge (*m/z*) ratio of 425 via electrospray ionization mass spectrometry analysis.

### SAM cleavage assays

The SAM cleavage assays contained, in a total volume of 0.5 ml, 25 mM Tris–HCL (pH 8.0), 5% glycerol, 16 μM Na_2_S_2_O_4_-free NifEN-B, NifEN-B^FeS^ or NifEN-B^FeSe^ and 10 μM SAM, [methyl-*d*_*3*_] SAM or allyl SAM. Assays were incubated at 25 °C for 30 min with intermittent mixing, before being terminated by filtration with Amicon Ultra 30,000 MWCO centrifugal filters. TFA was supplemented at a concentration of 0.14% to the filtered samples before the samples were analysed using a Thermo Scientific Dionex Ultimate 3000 UHPLC system with an Acclaim 120 C18 column (4.6 × 100 mm, 5-μm particle size). The column was equilibrated with 98% buffer A (50 mM KH_2_PO_4_, pH 6.6) and 2% buffer B (100% methanol). Following each 100-μl injection of sample, a linear gradient of 2–60% buffer B was applied over 20 min, followed by 10 min of isocratic flow with 60% buffer B and a linear gradient of 60–2% buffer B over 5 min. The buffer flow rate was 0.5 ml min^−1^ throughout each run and the column was maintained at 30 °C. The column was then equilibrated for 5 min with 2% buffer B before subsequent sample injections. The elution of SAM or allyl SAM cleavage products were monitored by ultraviolet at 254 nm.

### Acid-quenching experiments

A published method was adapted for the NifEN-B-dependent production of methanethiol, methylselenol or allylthiol^12^. First, excess Na_2_S_2_O_4_ was removed from NifEN-B via gel filtration with Sephadex G-25 fine resin that was equilibrated with an oxygen-free buffer containing 25 mM Tris–HCl (pH 8.0). Immediately following the removal of excess reductant, 4 nmol of NifEN-B or NifEN-B^FeSe^ was added to a sealed 300-μl glass vial, which contained 200 nmol SAM, [methyl-*d*_*3*_] SAM or allyl SAM in a total volume of 100 μl, as well as 100% argon at 2 psi in the headspace. The 100 μl reactions were then incubated for 30 min at 25 °C before being quenched by 25 μl of 1 M HCl.

To observe the formation of the volatile methanethiol, methane-*d*_*3*_-thiol, or methylselenol, the acid-quenched samples were incubated at 60 °C for 15 min and equilibrated to room temperature for 10 min before the entire headspace was injected by a gas-tight syringe onto a GC with flame photometric detection (SRI Instruments 8610C with a Restek Rt-XLSulfur column; 1 m, 0.75 mm ID) or a GC–MS (Thermo-Fisher Scientific Trace 1300 GC connected to a Thermo-Fisher Scientific ISQ QD single quadrupole mass spectrometry) with a Restek Rxi-1ms column (30 m, 0.32 mm ID, 4.0 μm df). The GC inlet and oven temperatures were maintained at 30 °C (for methanethiol or methane-*d*_*3*_-thiol analysis) or 50 °C (for methylselenol analysis), while the mass spectrometry transfer line and ion source were maintained at 250 °C. Total ion chromatograms were generated under SIM conditions in electron ionization mode, with *m/z*=47 for methanethiol detection, *m/z*=50 for methane-*d*_*3*_-thiol detection, and *m/z*=96 for methylselenol detection. These base peaks were selected based on characterization of standard samples (Sigma-Aldrich) under full scan conditions and comparison to those reported in the National Institute of Standards and Technology database. Approximately 50% of the maximum possible yield of methanethiol or methane-*d*_*3*_-thiol was detected in these experiments.

To observe the formation of allylthiol, the acid-quenched samples were incubated at 25 °C for 10 min before allylthiol was extracted into 100 μl of dichloromethane under anaerobic conditions. The DCM-extracted allylthiol was incubated at 60 °C for 15 min in sealed 300-μl glass vials and equilibrated to room temperature for 10 min before the entire headspace was injected onto a GC–MS (Thermo-Fisher Scientific Trace 1300 GC connected to a Thermo-Fisher Scientific ISQ QD single quadrupole mass spectrometry) with a Restek Rxi 1-ms column (30 m, 0.32 mm ID, 4.0 μm df). The inlet and oven temperatures of the GC were kept at 30 °C, while the mass spectrometry transfer line and ion source were maintained at 250 °C. Total ion chromatograms were generated for samples under SIM conditions (*m/z*=74) or full scan conditions (*m/z*=30–80) in electron ionization mode. The base peak was selected based on characterization of an allylthiol standard (Sigma-Aldrich) under full scan conditions and comparison with those reported in the National Institute of Standards and Technology database. Approximately 30% of the maximum possible yield of allylthiol was detected in these experiments.

## Additional information

**How to cite this article:** Wiig, J. A. *et al.* Refining the pathway of carbide insertion into the nitrogenase M-cluster. *Nat. Commun.* 6:8034 doi: 10.1038/ncomms9034 (2015).
